# Spontaneous Hepatic Rupture Complicating Preeclampsia and HELLP Syndrome: A Case Report

**DOI:** 10.1155/carm/4616669

**Published:** 2024-12-17

**Authors:** Bezza Kedida Dabi, Ahmed Siraj Mohammed, Fanta Asefa Disasa, Osias Tilahun Merga

**Affiliations:** ^1^Department of Obstetrics and Gynecology, Jimma Medical Center, Jimma University, Jimma, Ethiopia; ^2^Department of Surgery, Jimma Medical Center, Jimma University, Jimma, Ethiopia

**Keywords:** HELLP syndrome, hemoperitoneum, pregnancy, ultrasound

## Abstract

**Introduction:** Spontaneous hepatic rupture is a rare complication that occurs in pregnant mothers with HELLP syndrome, or preeclampsia with severe features, or eclampsia. The most common symptom of hepatic rupture/hematoma is right upper quadrant pain or epigastric pain, which is similar to the presentation of preeclampsia with severe features. Therefore, the absence of specific signs and symptoms leads to a diagnostic dilemma and a delay in management. The objective of this index study is to report available evidence on incidence, clinical presentation, pathophysiology, diagnosis, maternal and perinatal outcome, challenges, and best experiences in the management of hepatic rupture.

**Case History:** A 38-year-old G3P1A1 kaffa mother whose gestational age was 30 weeks plus 4 days from reliable last normal menstrual period had three ANCs. She presented with right upper quadrant pain of 3 days duration and severe global headache and blurring of vision of 5 days duration. Abdominal ultrasound showed a well-defined hyperechoic mass measuring 6 cm by 8 cm on the subcapsular area of the left lobe of the liver, as well as free intra-abdominal fluid. A diagnosis of ruptured hepatic subcapsular hematoma associated with preeclampsia with severe features was made, and an emergency laparotomy was done. Intraoperatively, 2500 mL of hemoperitoneum, a large hematoma (9 × 10 cm) on the left lobe of the liver, and active bleeding from the right lobe of the liver were found. Surgicell was applied to the actively bleeding site, and the right hepatic artery was ligated, along with perihepatic packing and a subhepatic drainage tube. Cesarean delivery was made to effect a delivery of a freshly dead male fetus weighing 1.4 kg. Despite this management, after 6 h of admission to the ICU, she passed away with a possible cause of death of multiorgan failure (liver, kidney, respiratory, and heart) secondary to underlying illness.

**Conclusion:** A high index of suspicion, multidisciplinary approach, and urgent laparotomy to secure hemostasis could prevent maternal death and perinatal loss due to hepatic rupture in preeclamptic mothers. The absence of specific signs and symptoms and a high case fatality rate mandate standardized protocols of management for hepatic rupture during pregnancy.

## 1. Introduction

Spontaneous hepatic rupture is a rare complication that occurs in pregnant mothers with HELLP syndrome (a group of symptoms that include hemolytic anemia, elevated liver enzymes, and thrombocytopenia) or preeclampsia with severe features or eclampsia. Preeclampsia is defined as a complex multisystem disease with the sudden onset of hypertension (blood pressure greater than or equal to 140/90 mmHg) after 20 weeks of gestation with proteinuria or maternal organ dysfunctions [1]. In the literature, the incidence of hepatic rupture ranges between 1:250,000 and 1:67,000 pregnancies, but in women with HELLP syndrome/preeclampsia/eclampsia, the incidence ranges between 0.9% and 2%, with one per 2000 deliveries [2, 3]. The most common symptom of hepatic rupture/hematoma is right upper quadrant pain or epigastric pain, which is similar to the presentation of preeclampsia with severe features [4]. Therefore, the absence of specific signs and symptoms leads to a diagnostic dilemma and a delay in management. Other clinical presentations include vomiting, shoulder pain, and sudden circulatory collapse in the presence of massive bleeding. Diagnosis is based on clinical presentation, ultrasound examination, and MRI imaging. However, most of the diagnoses are made based on intraoperative findings [2]. There is no standardized management protocol for hepatic rupture. Available options of management include perihepatic packing, ligation and embolization of the hepatic arteries, lobectomy, and liver transplant [2, 3, 5]. To the best of our knowledge, this fatal complication of preeclampsia has not yet been reported in Ethiopia. The objective of this index study is to report available evidence on incidence, clinical presentation, pathophysiology, diagnosis, maternal and perinatal outcome, challenges, and best experiences in the management of hepatic rupture through a review of literature.

## 2. Case Presentation

At Tepi General Hospital, a 38-year-old G3P1A1 kaffa mother at gestational age of 30 weeks plus 4 days, calculated from reliable last normal menstrual period, had undergone three antenatal care visits. She was told to have raised blood pressure 3 months back, at gestational age of 5 months, and she was on nifedipine 10 mg po daily. She was referred to Jimma Medical Center by a private Obstetrics and Gynecology clinic with the diagnosis of “preeclampsia with severe features in 3rd trimester pregnancy complicated with intrauterine fetal death and hypovolemic shock secondary to liver infarction on a background of preeclampsia complications.” She presented with right upper quadrant pain of 3 days duration and a severe global headache and blurring of vision of 5 days duration. She had a cessation of fetal movements of 1-day duration. Her previous pregnancies were one spontaneous vaginal delivery, which was uneventful, and one spontaneous abortion at 2 months of amenorrhea. She had no labor and rupture of membrane. She had no history of diabetes or hypertension other than raised blood pressure 3 months ago during her current pregnancy. She had no history of liver, renal, or cardiac illness. She had no history of trauma to her abdomen. Her familial history of liver disease was also unremarkable.

**Figure 1:**
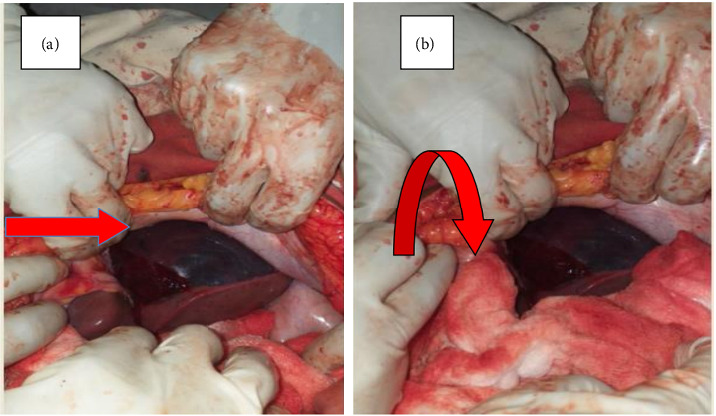
(a) Large hematoma is visible on the left lobe of the liver (straight arrow). (b) Perihepatic packing with sterile towel on the actively bleeding right lobe of the liver (curved arrow).

Upon physical examination, she was acutely sick looking (in pain). Her vital signs were blood pressure = 105/65 mmHg, pulse rate = 120 beats/minute, respiratory rate = 32 breaths/minute, and temperature = 36.3 C. Upon physical examination, she had pale conjunctiva. On abdominal examination, there was 30 week sized gravid uterus, longitudinal lie, and cephalic fetal presentation. Fetal cardiac activity was absent upon scanning by ultrasound. Laboratory tests indicated the blood group as O Rh D positive, with VDRL, hepatitis B surface antigen, and serostatus for HIV all showing negative results. White blood cell count of 17,000 m/L, hematocrit of 38%, platelet count of 32,900 m/L, liver enzymes with an alanine transaminase of 135 IU/L, aspartate transaminase of 124 IU/L, international normalized ratio of 0.52, prothrombin time (PT) of 11.4 s, activated partial PT of 30.1 s, renal function tests with creatinine of 1.2 mg/dL, urea of 42.2 mg/dL, and total and direct serum bilirubin were in the normal range. Abdominal ultrasound showed well-defined hyperechoic mass measuring 6 cm by 8 cm on the subcapsular area of the left lobe of the liver. Color Doppler imaging of the mass showed absent color flow within the mass. There was also free fluid in Morrison's pouch, paracolic gutters, and the pouch of Douglas. There were no visible intraparenchymal lesions/heterogeneous echoes of the liver. No imaging evidence of placental abruption was seen. The ultrasound impression was liver hematoma with intra-abdominal collection, likely hemoperitoneum. Intra-abdominal fluid was tapped, and it was frank blood. A diagnosis of ruptured hepatic subcapsular hematoma associated with preeclampsia with severe features was made, and the surgical department was consulted for emergency laparotomy. After 3 h of admission to the ward, laparotomy was decided; written consent was obtained from the patient and family (husband), 2 units of cross-matched blood were prepared, and she went to the operation theater accompanied by surgeons, a gynecologist, and other supportive health staff. Under general anesthesia, the abdomen was entered midline, and the intraoperative findings were as follows: 2500 mL of hemoperitoneum; a large hematoma (9 × 10 cm) on the left lobe of the liver (Figure 1); and active bleeding from the right lobe of the liver with a hematoma (5 × 6 cm) (Figure 1); specifically, segments VI and VII were bleeding, but segments II and III were not bleeding. Hemoperitoneum was sucked out; surgicell was applied to the actively bleeding site, ligation of the right hepatic artery was done to reduce blood flow to the actively bleeding right lobe of the liver, cauterization was made on the other small bleeding site of segment VII with perihepatic packing with sterile towels (Figure 1), and homeostasis was secured. Subhepatic drainage tube was put in. A cesarean section was done to effect a delivery of 1.4 kg freshly dead male neonate. During the surgery, she was transfused 3 units of whole blood, despite that her blood pressure dropped to the level of 85/50 mmHg, a PR of 150 beats/minute, and then she was put on continuous adrenaline infusion with the dose of 0.5 *μ*g per kilogram per minute (1 mL of 1:1000 solution) that was titrated based on the response. After two hours, surgery was completed, she was transferred to the surgical intensive care unit (ICU), transfused with an additional 2 units of blood, and resuscitated with IV normal saline, and the adrenaline drip continued. Despite the above management, she had no urine output. After 6 h of admission to the ICU, she passed away with a possible cause of death: multiorgan failure (liver, kidney, respiratory, and heart) secondary to underlying illness.

## 3. Discussion

We presented a case of spontaneous hepatic rupture complicating HELLP syndrome/preeclampsia, resulting in both maternal death and perinatal loss. HELLP syndrome was coined in 1982 to describe a syndrome characterized by hemolysis, elevated liver enzymes, and low platelets [6]. Although HELLP syndrome and disseminated intravascular coagulations share some common features, such as microangiopathic hemolytic anemia, thrombocytopenia, and elevated liver enzymes, there are several distinguishing factors. HELLP syndrome typically develops in pregnant women during the third trimester or postpartum period, often associated with preeclampsia or eclampsia, while DIC can be triggered by various underlying conditions, including sepsis, severe trauma, malignancies, obstetric complications, and severe infections, affecting individuals regardless of sex, age, or pregnancy status [7]. In HELLP syndrome, liver involvement is a prominent feature, characterized by elevated liver enzymes and complications such as subcapsular hematoma, hepatic rupture, or hepatic infarction, while in DIC, liver dysfunction is generally less pronounced and often results from the primary pathology or impaired blood flow and tissue hypoxia. Mild coagulopathy may be present in some cases of HELLP syndrome, while severe coagulation abnormalities are more characteristic of DIC, including elevated PT, activated partial thromboplastin time (aPTT), and decreased fibrinogen levels. The primary management strategy for HELLP syndrome involves prompt delivery of the fetus and supportive care to stabilize the mother's condition, while DIC treatment focuses on addressing the underlying cause, administering blood products and coagulation factors, and providing supportive care. Spontaneous hepatic rupture is rare, but it is a life-threatening complication associated with severe preeclampsia/HELLP syndrome. Hepatic rupture carries a high maternal and perinatal mortality of 39% and 42%, respectively [2]. Some reports of maternal death range from 40% to 80% based on the high complexity of management at different setups [8]. Before 1970, maternal death associated with hepatic rupture was 100% and decreased to 16.4% during 2001–2010 due to advanced resuscitation, intensive care medicine, and liver transplantation, especially in the developed world [9]. The majority of cases of hepatic rupture associated with preeclampsia occur in multiparous women, during 3^rd^ trimester pregnancy, and in their late 30s [2, 10], similar to our patient presentation. Hepatic rupture may occur because of underlying diseases like adenomas, malignancies, hemangiomas, coagulation disorders, and chronic hypertensive diseases; hepatic metastasis from choriocarcinoma; focal nodular hyperplasia; trauma; and infections like syphilis, malaria, and amebic liver abscess [11–13]. There is no evidence of underlying preexisting liver disease in our patient, as a liver autopsy examination was not done and her personal and familial histories were not remarkable for liver disease.

The pathogenesis of spontaneous hepatic rupture associated with preeclampsia/HELLP syndrome is not well known [14, 15]. The proposed pathogenesis is attributed to periportal hemorrhage and intravascular fibrin deposit that causes intrahepatic vascular congestion, leading to intrahepatic hemorrhage, rupture of the parenchyma, production of a subcapsular hematoma, and ultimately rupture of Glisson's capsule with intraperitoneal hemorrhage [16, 17]. Preeclampsia does not affect the liver primarily, and only 10% of patients had liver involvement [5]. The presenting symptoms and signs of hepatic rupture/hematoma are nonspecific, with the most common symptom being epigastric pain or RUQ pain, which is similar to the presentation of preeclampsia with severity features [12, 16, 17]. This leads to a diagnostic dilemma and delay in hepatic rupture management, and it is one of the contributing factors to our patient's maternal death and perinatal loss. A high index of clinical suspicion with emergency ultrasound or MRI is necessary for timely diagnosis and management of spontaneous hepatic rupture. In the index case, there was fetal loss at the diagnosis of hepatic rupture, and she had significant blood loss (2500 mL) by the time of laparotomy that contributed to multiorgan failure and maternal death. Lately, hepatic rupture presents with hypotension, shock, and hemoperitoneum [18], similar to the index case presentation.

Management of liver rupture requires a multidisciplinary approach with intensive hemodynamic monitoring [18, 19]. Observation is the preferred option of management in hemodynamically stable patients with liver hematoma [18, 19], but those with ongoing bleeding should undergo urgent laparotomy for damage control surgery. Surgical management involves perihepatic packing, hepatic artery ligation, surgicell application, collagen sponge, absorbable mesh, fibrin glue, argon laser, partial liver resection, or even liver transplantation [8, 15, 18, 19]. The index case was managed by ligation of the hepatic artery, perihepatic packing (Figure 1), surgicell application, and subhepatic drainage, as it has been reported to yield better outcomes [18, 20], and it was successful in achieving homeostasis. Even though there is intrauterine fetal death in the index case, cesarean section is the preferred mode of delivery to decrease the risk of hepatic rupture/hematoma formation with labor [21], similar to our patient management.

## 4. Conclusion

A high index of suspicion, multidisciplinary approach, and urgent laparotomy to secure homeostasis could prevent maternal death and perinatal loss due to hepatic rupture in preeclamptic mothers. Spontaneous hepatic rupture should always be considered as a potential diagnosis in pregnant mothers presenting with epigastric pain or RUQ pain followed by signs of hemodynamic instability.

## Data Availability

Data sharing is not applicable; no new data were generated.
